# Evidence of Progenitor Cell Lineage Rerouting in the Adult Mouse Hippocampus After Status Epilepticus

**DOI:** 10.3389/fnins.2020.571315

**Published:** 2020-09-18

**Authors:** Daniela M. S. Moura, Juliana Alves Brandão, Celia Lentini, Christophe Heinrich, Claudio M. Queiroz, Marcos R. Costa

**Affiliations:** ^1^Brain Institute, Federal University of Rio Grande do Norte (UFRN), Natal, Brazil; ^2^INSERM, Stem Cell and Brain Research Institute U1208, Univ Lyon, Université Claude Bernard Lyon 1, Lyon, France; ^3^Unité INSERM 1167, RID-AGE-Risk Factors and Molecular Determinants of Aging-Related Diseases, Institut Pasteur de Lille, University of Lille, U1167-Excellence Laboratory LabEx DISTALZ, Lille, France

**Keywords:** adult hippocampus, neurogenesis, astrogliogenesis, status epilepticus, fate-specification, kainic acid, pilocarpine, GABAergic interneurons

## Abstract

Cell lineage in the adult hippocampus comprises multipotent and neuron-committed progenitors. In the present work, we fate-mapped neuronal progenitors using Dcx-CreERT2 and CAG-CAT-EGFP double-transgenic mice (cDCX/EGFP). We show that 3 days after tamoxifen-mediated recombination in cDCX/EGFP adult mice, GFP+ cells in the dentate gyrus (DG) co-expresses DCX and about 6% of these cells are proliferative neuronal progenitors. After 30 days, 20% of GFP+ generated from these progenitors differentiate into GFAP+ astrocytes. Unilateral intrahippocampal administration of the chemoconvulsants kainic acid (KA) or pilocarpine (PL) triggered epileptiform discharges and led to a significant increase in the number of GFP+ cells in both ipsi and contralateral DG. However, while PL favored the differentiation of neurons in both ipsi- and contralateral sides, KA stimulated neurogenesis only in the contralateral side. In the ipsilateral side, KA injection led to an unexpected increase of astrogliogenesis in the Dcx-lineage. We also observed a small number of GFP+/GFAP+ cells displaying radial-glia morphology ipsilaterally 3 days after KA administration, suggesting that some Dcx-progenitors could regress to a multipotent stage. The boosted neurogenesis and astrogliogenesis observed in the Dcx-lineage following chemoconvulsants administration correlated, respectively, with preservation or degeneration of the parvalbuminergic plexus in the DG. Increased inflammatory response, by contrast, was observed both in the DG showing increased neurogenesis or astrogliogenesis. Altogether, our data support the view that cell lineage progression in the adult hippocampus is not unidirectional and could be modulated by local network activity and GABA-mediated signaling.

## Introduction

Generation and functional integration of new neurons occurs at discrete sites of the adult central nervous system (for review, see Lledo et al., 2006). In the adult rodent hippocampus, new granule cells (GCs) are constantly added to the dentate gyrus (DG) throughout life (van Praag et al., 1999), though at significantly lower levels in aging brains (Encinas and Sierra, 2012). Adult hippocampal neurogenesis contributes to learning and memory (for review, see Aimone et al., 2014) due to the unique responses of immature GCs to activity patterns entering the DG (Marín-Burgin et al., 2012). It also provides a powerful model to study the integration of newly generated neurons into preexisting matured neuronal circuits (Sailor et al., 2017).

Generation of new GCs in the adult hippocampus follows a stereotyped cell-lineage progression, with multipotent radial-glia like cell (RGCs, Type 1) progenitors generating glia-like non-radial (Type 2a) cells, which in turn give rise to neuronal-committed progenitors (Types 2b and 3), directly responsible for the production of post-mitotic neurons (Steiner et al., 2006). RGCs can transit from a quiescent to an activated state following external signals (Song et al., 2012; Andersen et al., 2014; Dumitru et al., 2017) and retain the ability to generate/differentiate into astrocytes (Bonaguidi et al., 2011; Encinas et al., 2011). However, it is largely believed that RGCs progress either through an astroglial-lineage producing new RGCs and astrocytes or a neuronal-lineage, comprising intermediate/type 2b and 3 progenitors, which terminates with the generation of new granule neurons (Steiner et al., 2004; Suh et al., 2007; Bonaguidi et al., 2011; Encinas et al., 2011; Dumitru et al., 2017). Although these intermediate progenitors can be genetically manipulated to generate glial cells (Braun et al., 2015), it remains unknown whether cell-extrinsic signals could also modulate their gliogenic potential.

Neuronal network activity affects cell proliferation and differentiation in the adult DGs and can be modulated by exposure to enriched environments (EE), running wheels, learning paradigms, stress and pathological conditions, such as epilepsy (Aimone et al., 2014). Among different neurotransmitter system modulating progenitor cell-lineage in the adult DG, gamma-aminobutyric acid (GABA) has been shown to promote neuronal differentiation and stem cell quiescence, whereas reduced GABA activity favors RGCs self-renewal and differentiation to astrocytes (Song et al., 2012; Dumitru et al., 2017). The effects of GABA on RGCs is mediated by the gamma-2 subunit of the GABA type A receptor expressed in RGCs (Song et al., 2012). However, activation of hilar interneurons also elicits a GABA_*A*_ receptor-sensitive response in Type 2 progenitors, causing depolarization and opening of voltage-dependent calcium channels (Tozuka et al., 2005). Calcium influx promotes NEUROD expression and leads to neuronal differentiation (Deisseroth et al., 2004). Thus, GABA signaling seems to act at different stages of the adult neural stem cell lineage toward generation of new DG granule cells.

In rodents, intrahippocampal injection of kainic acid (KA) has been largely used to model mesial temporal lobe epilepsy (MTLE) and is associated with hippocampal cell death, including degeneration of hilar GABAergic neurons (Ben-Ari, 1985; Bouilleret et al., 2000). Similarly, intrahippocampal injection of pilocarpine (PL) in rats leads to sustained status epilepticus, hippocampal cell death, and spontaneous seizures (Furtado et al., 2002, 2011; Castro et al., 2011). More recently, we and others have also validated this model of intrahippocampal PL injection to study MTLE in mice (de Lima et al., 2016; Moura et al., 2019). Interestingly, in both KA and PL animal models, alterations in the hippocampal adult neural stem cell lineage have been described using both BrdU-chasing (Parent et al., 1997; Scharfman et al., 2000; Heinrich et al., 2006; Ledergerber et al., 2006; Nitta et al., 2008) and genetic fate mapping of Glast-expressing cells (Andersen et al., 2014) or Nestin-expressing (Sierra et al., 2015). However, recent results from our group suggest that the effects of KA and PL on the hippocampal progenitor cell lineage can be divergent, with the first inducing astrogliogenesis and the second promoting neurogenesis (Moura et al., 2019).

We here hypothesized that KA and PL could differently affect the lineage progression of intermediate progenitors and that these effects could be correlated with opposing alterations in the GABAergic plexus of the DG. To evaluate this proposition, we used a Dcx-CreERT2 transgenic mouse line to fate map the lineage of DCX-expressing intermediate progenitors. Using intrahippocampal unilateral injections of KA and PL, we systematically compared the direct and indirect effects of these chemoconvulsants on the DCX-lineage. We show that DCX-expressing cells contribute a small proportion of astrocytes in the DG under physiological conditions. Still, increased neuronal network activity induced by local KA injection significantly shifts the Dcx-lineage toward an astrogliogenic fate. By contrast, similar increases in neuronal activity mediated by local PL injection or in the contralateral DG of both KA and PL injected animals are associated with enhanced neurogenesis, suggesting that local effects of KA rather than increased electrical activity *per se* are necessary for the switch of the DCX-lineage toward astrogliogenesis. Finally, we demonstrate a positive correlation between these effects on the Dcx-cell lineage progression and divergent alterations in the number of parvalbumin-expressing neurons, but not microglial activation within the DG.

## Materials and Methods

### Animals

All experiments performed involving animals were approved by the ethics committee for animals in the Federal University of Rio Grande do Norte (CEUA-UFRN) with protocol number 012/2016 conform guidelines from the regional council. For the present study, a total of 36 double-transgenic mice, with age between 8 and 12 weeks, were randomly assigned to the control or treatment [*status epilepticus* (SE) induced by KA or PL] group. Mice from the lineage DCX (DCX-CRE-ER^*T*2^ - Stem Cell Research, Helmholtz Center Munich) were crossed to mice from the reporter lineage GFP (CAG-CAT-GFP) to generate double-transgenic DCX-CreER^*T*2^:CAG-CAT-GFP. The genotypes of the mutants were confirmed by PCR analysis of genomic DNA. Animals had a mixed background from C57BL/6, Swiss and Agouti. Tamoxifen (TAM) treatment of these mice restricts CreER^*T*2^ expression to DCX-expressing progenitors and neuroblasts in the hippocampal subgranular zone (Zhang et al., 2010). It causes random excision between multiple pairs of lox sites, leading to the expression of the reporter sequence. To achieve sparse labeling, mice were given two injections of TAM (100 μg/g), in consecutive days. The sample size was calculated based on previous work considering power of 80% resulting in four animals per group. The animals were held under standard laboratory housing conditions with a light/dark cycle of 12 h each and free access to food and water. All efforts were made to minimize animal suffering.

### Intrahippocampal Kainic Acid, Pilocarpine and Retroviral Injections

Unilateral intrahippocampal injections were performed as previously described (Moura et al., 2019). Adult mice were anesthetized by inhalation using a mixture of isoflurane 1.5% (1l/min) and positioned in the stereotaxic frame. We injected 50 nl of KA (20 mM in PBS) or vehicle solution (sterile PBS) at the speed of 0.5 μl/min using a glass micropipette connected to a nanoinjector (Nanoject II, Drummond Scientific). Coordinates used were AP: 2.1; ML: 1.7; DV: 1.6 mm. Additional control experiments were performed by injecting KA in the proximity of the rostral migratory stream (AP: 1.7; ML: 0.7; DV: 1.6 mm). After injection, the micropipette was left still for 5 min to avoid liquid reflux. Following KA injection, SE develops within minutes. It was characterized by forelimbs’ clonic movements, rotations or immobility. In two animals, recordings of local field potentials confirmed the sustained epileptiform activity during SE. After 90 min of SE, mice received an intraperitoneal (i.p.) injection of diazepam (5 mg/kg) to relieve seizure activity. Mice were returned to their cage where they had free access to food and water and were monitored for weight loss or signs of pain. Only animals that had experienced SE after KA injection, and had confirmed granular cell dispersion in the histological analysis were kept for further analysis.

A similar protocol was applied for PL injection with small adaptations. Before anesthetizing the animal, methyl-scopolamine was injected (1 mg/kg, i.p.) to block peripheral reactions. Coordinates and drug delivery method were the same as KA, but the concentration of the solution and the volume applied were 700 μg/μL and 570 μl, respectively.

Intrahippocampal injection of the retrovirus encoding the DsRed fluorescent reporter under control of an internal CAG promoter (pCAG-IRES-DsRed; Heinrich et al., 2010) was performed following a similar protocol as for KA injection (coordinates AP: 2.1; ML: 1.7; DV: 1.6 mm). The viral suspension (0.5 μl) was slowly injected during 30–40 min to allow for an optimal diffusion of viral particles. Viral titers used for experiments were typically in the range of 10^6^–10^9^ transducing units/ml.

### Tamoxifen and BrdU Administration

A single tamoxifen (TAM, T-5648, Sigma-Aldrich) diluted in corn oil (10 mg/ml, Sigma) injection was administered i.p. (100 μg/g) 3, 7, or 30 days before perfusion to study the Dcx-lineage under physiological conditions. To explore the Dcx-lineage recombined after or before SE, two TAM (100 μg/g) injections were performed in consecutive days, respectively 1 week after or 5 weeks before intrahippocampal injections. For the recombination 24 h before SE induction, only a single TAM (100 μg/g) injection was performed 24 h before the intrahippocampal vehicle or KA injection. BrdU was administered either by i.p. injections (50 mg/kg every 12 h) or diluted in the drinking water (1 mg/ml continuously). Administration protocols used for each experiment are described in the results section and outlined in the main figures.

### Immunohistochemistry and Microscopy

Animals were deeply anesthetized with thiopental (100 mg/kg; i.p.) and transcardially perfused with saline 0.9% (10–15 min) and 4% paraformaldehyde (PFA) solution (15–20 min). Brains were removed and post-fixed in 4% PFA overnight at 4°C. Next, brains were cryoprotected in 30% sucrose solution for 24–48 h, and then frozen in isopentane with dry ice and stored at −80°C. Coronal sections (40 μm) were obtained using a cryostat, collected onto silanized slides, and stored at −20°C for posterior analysis.

Sections were incubated 24–48 h at 4°C in PBS 10 mM/TritonX 0,5%/Normal Goat Serum (NGS) 5% solution containing different combinations of the following primary antibodies. rabbit anti-GFAP (1:1000; DAKO), chicken anti-GFP (1:500; Aves Labs), rabbit anti-CTIP2 (1:500; Abcam), rabbit anti-DCX (1:1,000, Abcam); rat anti-BrdU (1:500, Abcam), mouse Anti-PV (1:1,000, Sigma); mouse anti-NEUN (1:1,000; Millipore); rabbit anti-IBA1 (1:1,000, Wako). After washing, sections were incubated 2 h at room temperature in the same solution containing appropriate secondary antibodies. After three washes, sections were stained with DAPI (1 μg/ml) and mounted using Aqua Poly/Mount (Polysciences, Inc.).

For BrdU and NEUN detection, slices were incubated with PFA 4% for 10 min and then pre-treated with sodium citrate solution 10 mM at 97°C for 15 min for antigen retrieval (Tang et al., 2007) before incubation with primary antibodies. When BrdU or NEUN detection was combined with other markers, we first completed immunohistochemistry for proteins that did not require antigen retrieval (GFP, CTIP2, GFAP, and DCX) and only then proceeded with pre-treatment and immunohistochemistry against BrdU or NEUN.

### Quantification and Statistical Analysis

Histological analyses were performed in serial sections sampled at 1/10 (i.e., one section every 400 μm in the anteroposterior axis). For quantitative analysis, we used Stereo investigator software to count cells and to measure the areas of analyses. The subdivisions of the DG - hilar region, molecular layer (ML) and granule cell layer (GCL) - were identified using the DAPI staining. For GFP quantification, four to five sections were analyzed per slide (sampling at 1.6–2.0 mm of the AP axis of the dorsal hippocampus), and the total number of GFP cells was counted in every section. To quantify co-labeling between GFP and DCX, BrdU, CTIP2, NEUN, or GFAP, we captured images using a confocal laser-scanning microscope (LSM 710, Carl Zeiss, Jena, Germany) aiming at all GFP+ cells present in the DG. *Z*-stacks were obtained using optimal intervals and pinhole set to one air unity. To quantify cell fates, the proportion of marker+/GFP+ (or marker+/DsRed+ for retrovirus injections) cells was normalized for the total number of GFP+ (or DsRed+) cells. For the characterization of GFP+ progenitors and their progeny, the percentages of marker+/GFP+/BrdU+ cells were normalized by the total of GFP+/BrdU+ cells.

For PV quantification, we counted PV+ positive cells spread along the hippocampus, including DG, CA3, and CA1. The slices were distributed from Bregma −1.3 to −3.4 mm. All the area was identified by DAPI staining. The number of PV cells was quantified in a total of three to five sections per slide using a Zeiss Imager M.2 Apotome microscope (Carl Zeiss) with a 20× objective and the Stereo investigator software. For IBA1 analyses, we sampled four areas of the DG (including the molecular layer, GCL and hilus) in four to five sections per animal from each experimental group (control, PL and KA). Images were acquired using LSM710 confocal microscope and 40× objective. Sections analyzed were distributed from Bregma −1.3 to −2.5 mm. Morphological analyses of microglial cells to detect resting and activated cells were performed using ImageJ v1.4 with MorphoLibJ integrated library and plugin (Schindelin et al., 2012; Legland et al., 2016) as previously described (Davis et al., 2017). Briefly, we first applied a grayscale attribute opening filter (area minimum: 50 pixels; connectivity: 8) to each maximum projection of IBA1 staining. Next, to separate microglia soma from processes, we used an opening morphological filter (1-pixel radius octagon) before a maximum entropy threshold to segment the microglia soma from the image background. We double-checked the contouring of soma and completed measuring soma area with ImageJ Analyze Particles function. Classification of microglia was made based on the morphology of the cell body, and processes and area measurement were used for posterior analysis. Activated microglia was defined as IBA1+ cells with enlarged cell body and processes. The proportion of IBA1+ aMGCs among total IBA1+ cells was quantified in all three DG layers: molecular layer; GCL; and hilus.

Values are expressed as mean ± standard error of the mean (SEM). For all comparisons, one-way or two-way ANOVA (treatment group and hemisphere as factors) were performed, followed by *post hoc* tests, whenever appropriate. Statistical tests were performed using GraphPad Prism version 6. The confidence interval is 95%. Differences were considered statistically significant at ^∗^*p* < 0.05, ^∗∗^*p* < 0.01, ^∗∗∗^*p* < 0.001, ^****^p < 0.0001.

## Results

### Dcx-Lineage in the Adult Hippocampus Encompasses Astrocytes

**FIGURE 1:**
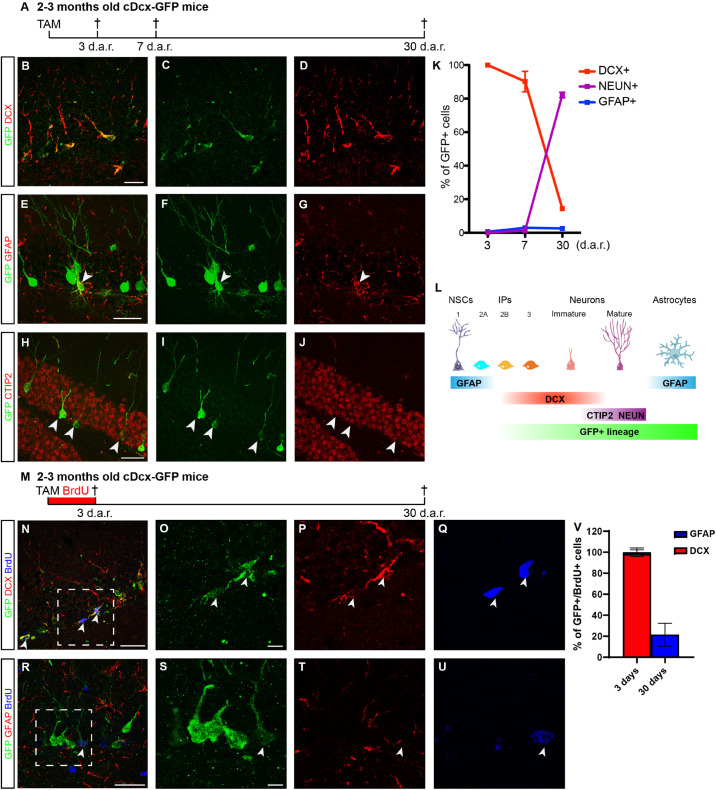
Dcx-lineage comprises mostly neurons and a small fraction of astrocytes. **(A)** Timeline presenting timepoints (d.a.r. – days after recombination) in which animals were fixed (crosses) for immunohistochemistry and quantification of GFP+ cells after TAM administration. **(B–J)** Coronal sections of the hippocampus of cDCX/EGFP animals immunolabeled for GFP (green), DCX, GFAP, or CTIP2 (red) 3, 7, or 30 d.a.r. Observe that GFP expression is restricted to small DCX+ cells in the SGZ 3 d.a.r. **(B–D)**. **(E–G)** Example of a GFP+/GFAP+ cell showing morphologies of astrocytes 30 d.a.r. (arrowhead). **(H–J)** Examples of GFP+/CTIP2+ cells showing mature granule cell morphology 30 d.a.r. (arrowheads indicate some of these cells). **(K)** Graphic showing the quantification of GFP cells expressing makers of different cell populations (*n* = 4–5 animals per timepoint). **(L)** Schematic representation of adult hippocampal neurogenesis showing markers in each phase of cell development used in this study. NSCs – Neural stem cells (type 1); IPs – Intermediate progenitors (types 2a, 2b, and 3). **(M)** Timeline presenting experimental protocol from group receiving TAM injection followed by 3 days of continuous BrdU treatment in the drinking water. Animals were perfused 3 or 30 days after recombination (d.a.r.). **(N–Q)** Expression of DCX in GFP+/BrdU+ cells 3 d.a.r. **(R–U)** 30 days after Dcx-mediated recombination, GFP+/BrdU+ are rare possibly due to cell death of newly generated cells. **(V)** Graphic showing the quantification of GFP+/BrdU+ cells expressing DCX or GFAP. (*n* = 5 animals in “3 days” group; *n* = 3 in “30 days” group). Scale bars: 20 μm.

Cell lineage in the adult hippocampus comprises multipotent (Types 1 and 2a) progenitors and neuron-determined (Types 2b and 3) progenitors (Steiner et al., 2006). To label and follow the latter, we generated double-transgenic mice crossing Dcx-CreERT2 (Zhang et al., 2010) and CAG-CAT-EGFP mice (Nakamura et al., 2006), hereafter referred to as cDcx/GFP. Animals were killed 3, 7, or 30 days after tamoxifen (TAM) treatment (Figure 1A). Using confocal microscopy, we independently analyzed the co-localization between GFP and DCX (intermediate progenitors and immature neurons), GFAP (astrocytes), CTIP2 (immature and mature granule cells) or NEUN (small fraction of maturing and mature granule cells (Figures 1B–J) (Steiner et al., 2006; Simon et al., 2012). Three days after TAM administration, 97% of GFP+ cells in the dentate gyrus (DG) co-expressed the protein DCX and were either cells with short horizontal processes located in the subgranular zone (SGZ) or cells with radially oriented processes located in the inner half of the granular layer (Figures 1B–D,K; *n* = 5 animals; 106 GFP+/DCX+ out of 108 GFP+ cells), as expected for type 2 progenitors and immature neurons, respectively (Brown et al., 2003; Jagasia et al., 2009). In contrast, only 0.5% of GFP+ cells co-stained for GFAP in the hilus (Figure 1K; *n* = 5 animals; 1 GFP+/GFAP+ cell out of 198 GFP+ cells). Seven days after recombination, 90% of GFP+ cells occupied the inner half of the granular layer, showed morphologies reminiscent of immature granule neurons and co-expressed DCX (Figure 1K; *n* = 4 animals; 368 GFP+/DCX+ out of 415 GFP+ cells). A significant proportion of these GFP+/DCX+ cells also expressed CTIP2 (data not shown), but only 1–2% of GFP+ cells already expressed NEUN (Figure 1K), a protein associated with the maturation of adult-born neurons in the DG. Interestingly, we also observed at this time-point a small but consistent population of GFP+ cells expressing the astrocytic protein GFAP (Figures 1E–G,K; *n* = 4 animals; 13 GFP+/GFAP+ out of 476 GFP+ cells). Thirty days after recombination, 15% of GFP+ cells still expressed DCX and 82% of the recombined cells displayed typical morphology of mature granule neurons and expressed NEUN and CTIP2 (Figures 1H–K; *n* = 4 animals; 26 GFP+/DCX+ and 142 GFP+/NEUN out of 172 GFP+ cells). At this time-point, we could still detect about 3% of GFP+ cells expressing GFAP (Figure 1K; *n* = 4 animals; 4 GFP+/GFAP+ out of 131 GFP+ cells). Altogether, these data confirm previous observations indicating that the Dcx-lineage in the adult hippocampus mostly encompasses newly generated granule neurons (Zhang et al., 2010), but unexpectedly contains a small percentage of astrocytes at days 7 and 30.

To probe whether GFP+ astrocytes could be derived from Dcx-expressing progenitors in the DG, we treated cDcx/GFP adult animals with tamoxifen followed by 3-days continuous BrdU administration in the drinking water (Figure 1M). A subset of animals was killed immediately after BrdU administration (therefore, 3 days after tamoxifen), and the remaining animals were allowed to survive for an additional 27 days (30 days after tamoxifen). We found that 94% of recombined GFP+ cells did not incorporate BrdU during the 3-days treatment (503 GFP+/BrdU− out of 534 GFP+ cells). In contrast, the remaining 6% of GFP+ cells were labeled with BrdU, indicating that only a small fraction of recombined cells in the DG of cDcx/GFP mice are type 2b/3 intermediate progenitors, in agreement with the low proportion of DCX+ proliferating cells in the DG of adult mice (Liu et al., 2015). Among those cells, 94% were also DCX+ (Figure 1V; *n* = 5 animals; 29 GFP+/BrdU+/DCX+ out of 31 GFP+/BrdU+ cells). In contrast, only in 1 out of 5 animals analyzed we could detect a single GFP+/BrdU+ cell co-expressing GFAP (Figure 1V; *n* = 5 animals; 1 GFP+/BrdU+/GFAP+ out of 51 GFP+/BrdU+ cells), indicating that unspecific recombination of GFAP-expressing progenitors in the DG of cDcx/GFP mice following our TAM administration protocol is unlikely. Even considering the total population of BrdU+ cells in the DG that did not express GFP, only 4% of cells co-expressed GFAP, which is in accordance with the low number of Type 1/2a progenitors in the DG (Encinas et al., 2011). Notably, however, 30 days after Dcx-mediated recombination, we observed that up to 19% of GFP+/BrdU+ cells co-expressed the astrocyte protein GFAP (Figure 1; *n* = 3 animals; 5 GFP+/BrdU+/GFAP+ out of 27 GFP+/BrdU+ cells). Altogether, these observations may suggest that at least some of GFAP+/BrdU+/DCX+ cells observed 3 days after recombination are DCX+ Type 2b/3 neuronal progenitors that retain the ability to generate astrocytes.

### Divergent Effects of Kainic Acid and Pilocarpine on the Dcx-Lineage

**FIGURE 2:**
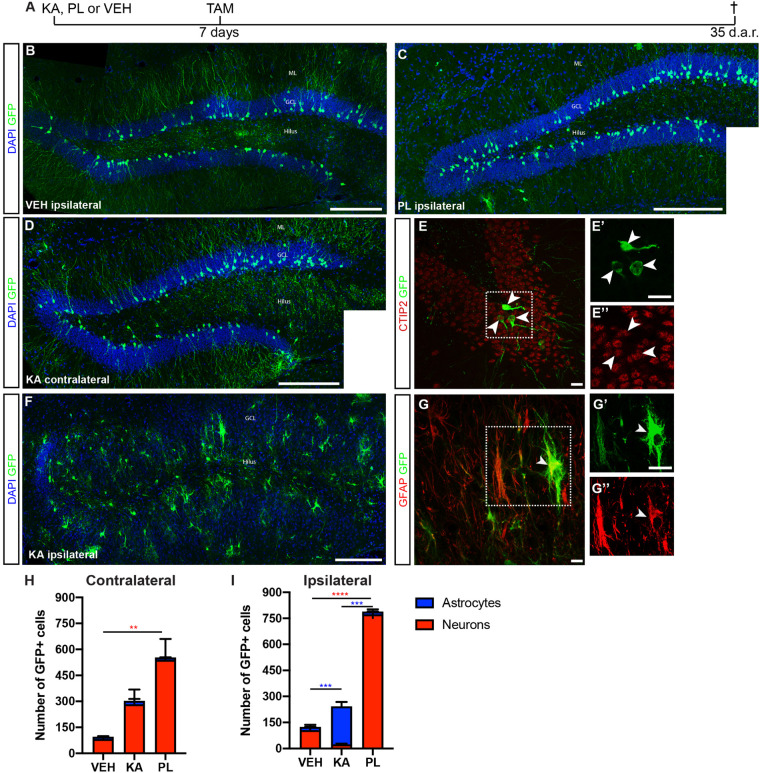
Kainic acid and pilocarpine have opposite effects in Dcx-lineage progression. **(A)** Timeline of the experimental protocol used to fate map the Dcx-lineage recombined 7 days after kainic acid (KA), pilocarpine (PL) or vehicle (VEH) intrahippocampal injection. **(B–F)** Coronal sections of the hippocampus of cDCX/EGFP animals immunolabeled for GFP (green), GFAP or CTIP2 (red). Mosaic composition of VEH **(B)** and PL **(C)** ipsilateral side and KA contralateral hippocampus **(D)** showing GFP+ granule cells with typical morphology. **(E)** Confocal image of contralateral hippocampus in KA group showing the expression of CTIP2 in GFP+ granule cells in single plane *z*-projection images. **(F)** Mosaic composition of ipsilateral hippocampus of KA injected animals showing severe GCD and GFP+ cells with morphologies of reactive astrocytes. **(G)** Confocal image of ipsilateral DG showing the expression of GFAP in GFP+ cells in single plane *z*-projection images. ML, molecular layer; GCL, granular cell layer. **(H,I)** Quantification of the total number of GFP+ cells differentiating into granule neurons (red bars) or astrocytes (blue bars) in the contralateral and ipsilateral sides. (*n* VEH = 3; *n* KA = 5; *n* PL = 3; two-way ANOVA followed by Tukey’s multiple comparisons: **Padj < 0.01; ***Padj < 0.001; ****Padj < 0.0001). Graphic shows mean ± SEM of animals with same number of sections analyzed. Scale bars: 100 μm **(B–D,F)** and 20 μm **(E,G)**.

The observation that a small subset of neuronal progenitors in the Dcx-lineage could retain the potential to generate astrocytes prompt us to evaluate whether changes in network excitability could interfere with the lineage progression of these progenitors, as it has been shown for other systems (Lin et al., 2017). Previous work from our group has shown that unilateral intrahippocampal injection of the chemoconvulsants KA or PL in adult cDcx/GFP mice induced long-lasting bilateral epileptiform discharges, behavioral seizures indicative of *status epilepticus* (SE), and changes in the hippocampal neurogenesis/astrogliogenesis (Moura et al., 2019). To further characterize these alterations induced by KA and PL, we fate-mapped the Dcx-lineage in cDcx/GFP animals following SE induction (Figure 2 and Supplementary Figure S1). TAM was administered 1 week after chemoconvulsant injection, and the phenotype of GFP+ cells was evaluated 4 weeks later (Figure 2). According to previous data in the literature suggesting that cell proliferation/survival could be affected by KA or PL (Parent et al., 1997; Scharfman et al., 2000; Kralic et al., 2005; Heinrich et al., 2006; Moura et al., 2019) we observed an overall increase in the number of GFP+ cells in both hippocampi of KA- and PL-treated animals (Figures 2H,I). However, while in both ipsi and contralateral DG of PL animals a significant increase in the number of GFP+ neurons could be detected (Figures 2H,I), this was not the case in KA animals. In these animals, a significant increase in the number of GFP+/CTIP2+ neurons could be observed only in the contralateral side compared to controls (Figures 2D,E,H). By contrast, a reduced number of neurons and a significant increase in the number of GFP+/GFAP+ astrocytes were observed in the ipsilateral side compared to both control and PL-treated animals (Figures 2F,G,I and Supplementary Figure S2). These observations suggest that the bilateral increased electrical activity elicited by PL- or KA-injection in the hippocampus may be permissive to either neurogenesis or astrogliogenesis depending on other local effects of those chemoconvulsants.

To rule out the possibility that KA injection could induce activation of the Dcx promoter directly in astrocytes leading to CRE-ERT expression and subsequent TAM-mediated recombination, we injected KA in the striatum, close to the rostral migratory stream (RMS) of cDcx/GFP adult mice, followed by a single TAM injection 7 days later, and analyzed the expression of GFP in reactive astrocytes after 35 days. In contrast to the hippocampus, no GFP+/GFAP+ cells were observed in the striatum or olfactory bulb (OB; Supplementary Figure S3), supporting the notion that KA *per se* does not activate the Dcx promoter in astrocytes of cDcx/GFP double-transgenic animals. Also consistent with the interpretation that Dcx-promoter is active only in the neuronal lineage of the RMS-OB system, we observed that GFP+ cells differentiated into OB granular and peri-glomerular interneurons 35 days after TAM (Supplementary Figure S3).

### Kainic Acid Induces Astrogliogenesis From Dcx-Progenitors Recombined 1 Day Before Status Epilepticus

**FIGURE 3:**
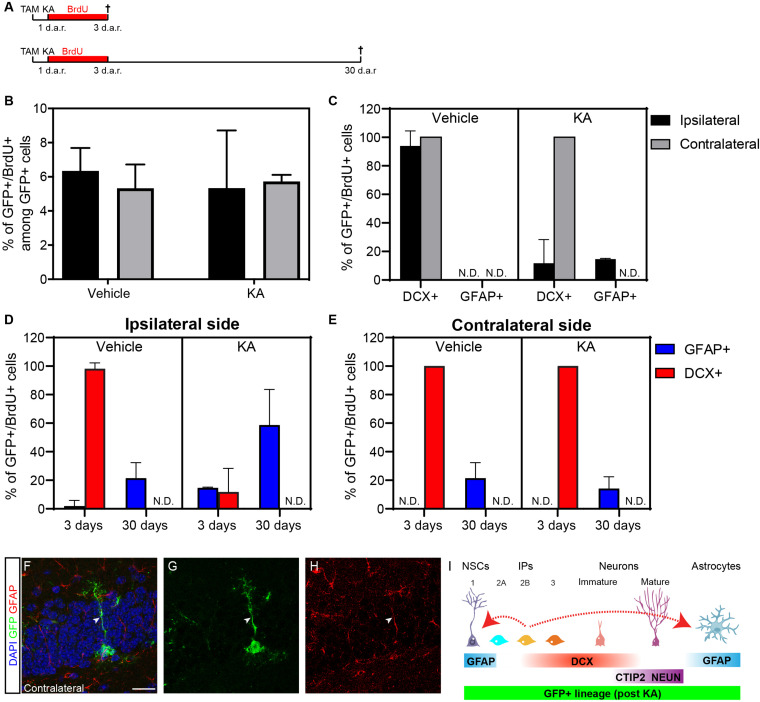
Kainic acid injection enhances astrogliogenesis from previously recombined DCX-cells. **(A)** Timeline representing experimental protocols in which one tamoxifen injection was administered 1 day before SE induction with KA. Animals received BrdU in drinking water during 3-days and were perfused right after this period, or 30 days after recombination (d.a.r.). **(B,C)** Quantification of proliferative GFP+ cells after 3 days **(B)**, and expression of DCX or GFAP among these cells **(C)** on ipsilateral and contralateral side of vehicle and KA injected animals. **(D,E)** Quantification of proliferative GFP+ cells after 3 and 30 days in ipsilateral side **(D)** and contralateral **(E)** expressing DCX or GFAP. **(F–H)** Example of GFP+/GFAP+ cell (RGL) in the SGZ of KA injected cDCX/EGFP animals 3 days after recombination. **(I)** Schematic representation of the fate switches (dotted red arrow) observed in the Dcx-lineage after KA injection.

Next, to unambiguously exclude possible influences of KA on the activity of the Dcx promoter in the hippocampus, we recombined Dcx-expressing cells before SE induction. To that, cDcx/GFP mice received tamoxifen 1 day before intrahippocampal injection of KA or vehicle (Figure 3). Animals also received BrdU in the drinking water for three consecutive days to label proliferating cells and were killed immediately after this period or 30 days after KA. We observed that the proportion of GFP+ cells undergoing cell proliferation during the 3 days BrdU-treatment was similar between control and KA animals (Figure 3B; *n* vehicle = 5 animals, 268 GFP+ cells in the contralateral and 281 in the ipsilateral side; *n* KA = 3 animals, 249 GFP+ cells in the contralateral and 759 in the ipsilateral side). However, while in both hippocampi of control animals and in the contralateral side of KA-treated animals, virtually all GFP+/BrdU+ cells co-expressed DCX, about 14% of GFP+/BrdU+ cells already expressed GFAP in the ipsilateral side of KA-injected mice at this time point (Figure 3C; *n* vehicle = 5 animals, 21 GFP+/BrdU+ cells in the contralateral and 18 in the ipsilateral side; *n* KA = 3 animals, 14 GFP+/BrdU+ cells in the contralateral and 48 in the ipsilateral side). This fraction of GFP/GFAP/BrdU cells in the KA-ipsilateral side further increased to about 58% after 30 days (Figure 3D; *n* vehicle = 4 animals, 18 GFP+/BrdU+ cells in the contralateral and 12 in the ipsilateral side; *n* KA = 3 animals, 25 GFP+/BrdU+ cells in the contralateral and 46 in the ipsilateral side). Also, despite the complete absence of GFP+/GFAP+/BrdU+ at day 3 in control animals and in the KA-contralateral side, these cells could be observed at small but consistent frequencies 30 days after recombination (Figures 3D,E). Altogether, these observations confirm that astrocytes are encompassed within the lineage of Dcx-expressing Type 2b/3 cells, and that generation of new astrocytes from Type 2b/3 cells depends on cell proliferation.

The generation of astrocytes from Dcx-expressing progenitors (Types 2b and 3) may suggest that those cells are bipotent or may reverse to a more primitive stage in the lineage when cells retain astrogliogenic potential (Bonaguidi et al., 2011; Encinas et al., 2011). To directly address this second possibility, we set out to investigate whether cells within the Dcx-lineage could adopt hallmarks of Type 1, radial glia-like (RGL) progenitors. In both untreated, vehicle- and PL-injected animals we were unable to detect any GFP+/GFAP+ RGL cells in the SGZ at all time points analyzed (3, 7, or 30 d.a.r). In contrast, we found a small number of GFP+/GFAP+ RGL cells in animals treated with KA both in the ipsilateral and contralateral hippocampus (Figure 3G; *n* = 3 animals; 8 GFP+/GFAP+ RGL cells out of 387 GFP+ cells). These observations could suggest that Type 2b/3 Dcx-expressing progenitors are not fully committed to differentiate into neurons but may rather return to more primitive stages in the lineage and resume multipotency under non-physiological conditions.

### Progenitors in the Dcx-Lineage Retain the Ability to Generate Neurons and Astrocytes 1 Month After Recombination

**FIGURE 4:**
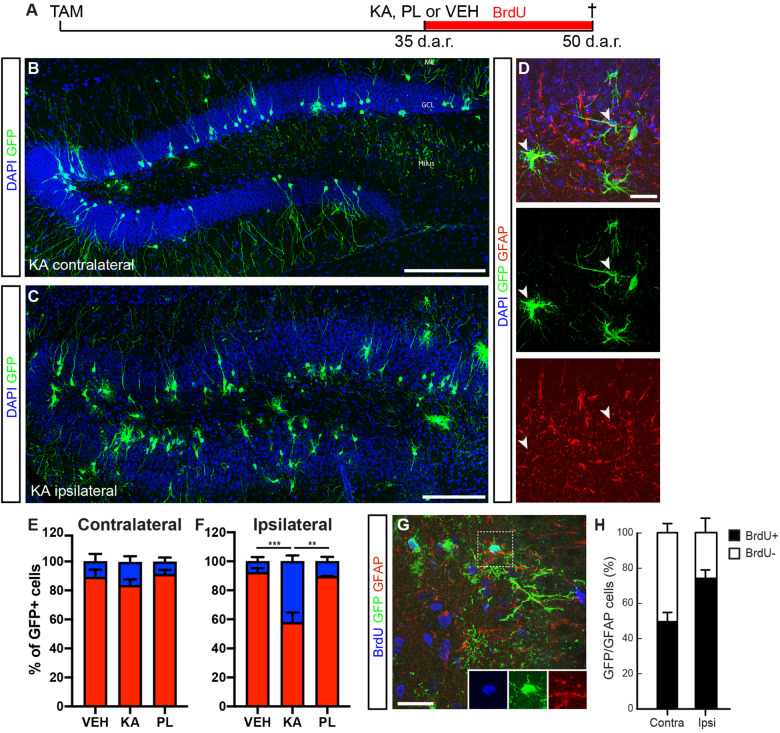
DCX-cells retain the capacity to proliferate and generate astrocytes after 1 month. **(A)** Timeline of the experimental protocol used to fate map the Dcx-lineage recombined 35 days before kainic acid (KA), pilocarpine (PL) or vehicle (VEH) intrahippocampal injection. Animals were treated with BrdU in the drinking water during the whole period after KA and were perfused 2 weeks later. **(B,C)** Coronal sections of the hippocampus of cDCX/EGFP animals immunolabeled for GFP (green). Mosaic image of the KA contralateral DG shows the majority of GFP+ cells with typical neuronal morphologies **(B)**. Mosaic image of the KA ipsilateral DG shows GCD and many GFP+ cells with morphologies of astrocytes **(C)**. **(D)** Coronal sections of the hippocampus of cDCX/EGFP animals immunolabeled for GFP (green) and GFAP (red) showing GFP+/GFAP+ astrocytes (arrowheads) in the ipsilateral DG. **(E,F)** Quantification of GFP+ cells differentiating into neurons (red bars) or astrocytes (blue bars) in the contra **(E)** and ipsilateral **(F)** DG of controls and KA-treated animals. **(G)** Coronal sections of the hippocampus of cDCX/EGFP animals immunolabeled for GFP (green), GFAP (red), and BrdU (blue). Dashed box indicate a GFP+/GFAP+/BrdU+ astrocyte. Individual channels are shown in the right bottom corner. **(H)** Quantification of BrdU+ and BrdU− GFP+/GFAP+ cells the contra and ipsilateral DG of KA injected animals. ML, molecular layer; GCL, granular cell layer. Scale bars: 50 μm **(B,C)** and 20 μm **(D,G)**. [*n* vehicle = 4; *n* KA = 4; *n* PL = 3; two-way ANOVA followed by Tukey’s multiple comparisons: **Adjusted *p*-value (KA vs. PL) = 0.0018; ***Adjusted *p*-value (KA vs. VEH) = 0.0008)].

The interesting observation that Dcx-expressing progenitors can regress to previous stages of the lineage and generate astrocytes prompted the question as to which extent this potential could be observed in progenitors recombined at earlier time-points before KA injection. To address this question, we injected tamoxifen in cDcx/GFP adult mice 35 days before intrahippocampal injections of chemoconvulsants (Figure 4). Fifteen days after KA treatment, we observed a significant increase in the proportion of astrocytes among GFP+ cells in the KA-ipsilateral side as compared to the contralateral KA side, PL- and vehicle-injected animals (Figures 4B,C,E,F; *n* vehicle = 3 animals, 65 astrocytes out of 974 GFP+ cells in contralateral and 57 out 988 GFP+ cells in ipsilateral side; *n* PL = 3 animals, 34 astrocytes out of 474 in contralateral and 38 out of 363 GFP+ cells in ipsilateral side; *n* KA = 4 animals; 160 astrocytes out of 1,053 in contralateral and 405 out of 1,170 GFP+ cells in ipsilateral side). Yet, and consistent with our previous findings, a small population of GFP+/GFAP+ cells could be observed in both hippocampi of PL and control animals, as well as in the contralateral-KA side (Figures 4E,F). These observations suggest that Dcx-expressing progenitors recombined 5 weeks before KA injection retain their ability to proliferate and generate astrocytes. In fact, when the same experiment was performed with the additional treatment of animals with BrdU for the whole period after KA-injection, we observed that most GFP+ astrocytes in the KA-ipsilateral side incorporated BrdU (Figures 4G,H; *n* KA = 3 animals; 55 GFP+/GFAP+/BrdU+ out of 77 GFP+/GFAP+ cells), indicating that despite the low numbers of progenitor cells recombined within the Dcx-lineage, some are still active 35 days after recombination and retain the potential to generate astrocytes. According to this interpretation, we also observed some GFP+/GFAP+ RGL cells in the SGZ of these animals (Supplementary Figure S4).

Next, we followed an independent approach based on retroviral-mediated labeling of fast-dividing types 2/3 intermediate progenitors in the hippocampal subgranular zone (Supplementary Figure S5). To this end, adult mice were initially injected with a retrovirus encoding a DsRed fluorescent reporter 1 month before receiving intrahippocampal injection of KA or saline for controls, and the identity of DsRed+ cells was analyzed a month later (Supplementary Figure S5A). Interestingly, we could also observe that injection of KA, 4 weeks after retroviral infection, shifted the lineage toward a gliogenic fate (Supplementary Figures S5B–D; KA: 567 glia out of 1,045 DsRed+ cells in the ipsilateral side, *n* = 7 mice; vehicle: 21 astrocytes out of 224 DsRed+ cells in the ipsilateral side, *n* = 4 mice). Altogether, these data using two independent strategies to fate-map the progeny of type2b/3 progenitors further support our interpretation that these cells retain astrogliogenic potential.

### Loss of Parvalbumin-Expressing Interneurons Correlates With Increased Astrogliogenesis

**FIGURE 5:**
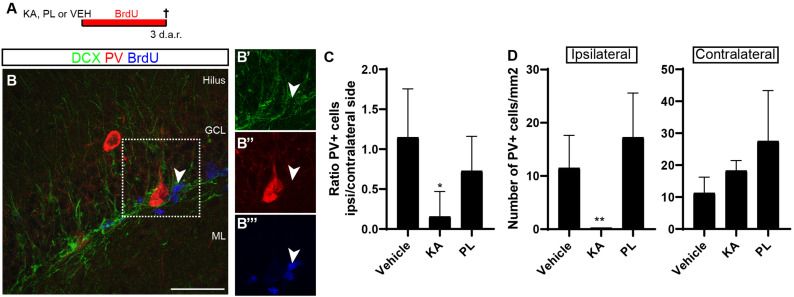
Parvalbuminergic plexus degenerates in the ipsilateral side of KA injection. **(A)** Timeline of the experiment. Animals received unilateral intrahippocampal injection of KA, PL or VEH followed by continuous BrdU administration in the drinking water for 3 days before perfusion. **(B)** Coronal section of the hippocampus of VEH animals immunolabeled for DCX (green), PV (red), and BrdU (blue). Note the presence of PV+ interneurons in the granule cell layer (GCL). Observe process of PV+ cell in close contact with proliferating BrdU+/DCX+ cell (arrowhead) in the subgranular zone (SGZ). BrdU administration was performed for three consecutive days in the drinking water before perfusion. Scale bar: 50 μm. **(C)** Ratio of PV+ cells in the ipsi and contralateral sides of the hippocampus in different treatment groups. **(D)** Quantification of the number of PV+ interneurons per mm^2^ of the dentate gyrus 3 days after injection of saline, KA or PL. [*n* vehicle = 4; *n* KA = 4; *n* PL = 4; Statistics: **(B)** ANOVA *F*_(__2_,_9__)_ = 4.529; *p* = 0.0436; Tukey’s multiple comparisons test DF = 9; Adjusted *p*-value (control vs. KA) = 0.0363; **(C)** ANOVA *F*_(__2_,_9__)_ = 8.347; *p* = 0.0089; Tukey’s multiple comparisons test DF = 9; Adjusted *p*-value (KA vs. PL) = 0.0077].

To get some insights about the possible mechanisms controlling the switch from neurogenesis to gliogenesis in the Dcx-lineage observed upon KA treatment, we evaluated the effects of this drug and PL on the parvalbuminergic plexus of the DG. GABA activity mediated by parvalbumin (PV)+ interneurons promotes stem cell quiescence and neuronal differentiation in the adult hippocampus (Song et al., 2012; Dumitru et al., 2017). We hypothesized that KA and PL, by activating kainate and muscarinic receptors, respectively, could have different effects on the survival of PV+ interneurons. To test this possibility, we analyzed the hippocampus of adult mice 3 days after vehicle, KA or PL intrahippocampal injection (Figure 5). According to previous data (Song et al., 2012), we observed a complex parvalbuminergic plexus within the subgranular zone, originating from PV+ cells in the hilus and in close relation with type 2b/3 BrdU+/DCX+ progenitors (Figure 5A). Next, we quantified the total number of PV+ interneurons and found that the ipsi/contralateral ratio of PV+ cells in the DG was significantly reduced in KA-injected animals, but not in animals treated with PL (Figure 5B; *n* = 3 animals per group, 4–5 coronal sections from each animal). Interestingly, whereas the total number of PV+ cells per millimeter square in the ipsilateral DG of control and PL animals was similar, it dropped close to zero in KA-injected animals (Figure 5C), in agreement with previous studies (Bouilleret et al., 2000). Conversely, the number of PV+ cells in the contralateral KA-side and in both DG of PL-injected animals was similar to vehicle-treated animals (Figure 5C). A similar pattern was also observed when we counted all PV-expressing cells in the whole hippocampus, including CA1/CA3 regions (Supplementary Figure S6), suggesting that the almost complete loss of PV+ interneurons is restricted to the ipsilateral hippocampus of KA-treated animals. Thus, the region where Dcx-progenitors generate astrocytes (ipsilateral KA-injected DG) shows both an increase of electrical activity (Supplementary Figure S1) and overall reduction in the number of PV+ interneurons in the DG.

### Microglia Activation Is Associated Both With Increased Hippocampal Neurogenesis and Astrogliogenesis

**FIGURE 6:**
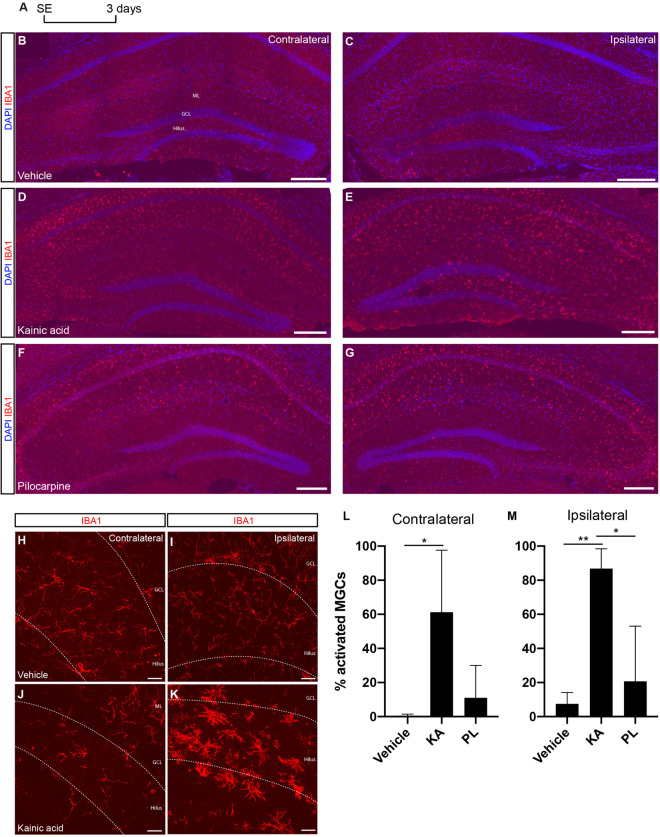
Microglia activation is stronger in the ipsilateral side of KA injection. **(A)** Timeline of experimental procedure to quantify Iba1+ cells. **(B,C)** Microglial cells in control group labeled with anti-Iba1 antibody and showing resting morphologies. **(D,E)** Increased signal for anti-IBA1 antibody in the KA group, indicating bilateral activation of microglia in the CA1/CA3 regions and in the ipsilateral DG hilus and molecular layer (not quantified). **(F,G)** Increased signal for anti-IBA1 antibody in the PL group, indicating bilateral activation of microglia in the CA1/CA3 regions (not quantified). **(H–K)** Higher magnification of the DG in vehicle **(H,I)** and KA **(J,K)** animals showing the morphological changes of microglial cells in the ipsilateral KA group. **(L,M)** Quantification of activated microglia in the ipsi or contralateral DG of vehicle-, KA- and PL-treated animals. [*n* control = 3; *n* KA = 2; *n* PL = 3; Statistics: **(L)** ANOVA *F*_(__2_,_5__)_ = 5.819; *p* = 0.0495; Tukey’s multiple comparisons test DF = 5; Adjusted *p*-value (control vs. KA) = 0.0486; **(M)** ANOVA *F*_(__2_,_6__)_ = 13.24; *p* = 0.0063; Tukey’s multiple comparisons test DF = 6; Adjusted *p*-value (vehicle vs. KA) = 0.0072; (PL vs. KA) = 0.0166].

Impaired inflammatory response has been described in rodent models of MTLE (Heinrich et al., 2006; Sierra et al., 2015; Moura et al., 2019), and may negatively affects neurogenesis (Monje et al., 2003; Sierra et al., 2015). To evaluate whether unilateral KA and PL intrahippocampal injections could elicit different degrees of inflammation in the ipsi and contralateral sides, we quantified the total number of resting and activated microglial cells in the DG, including the hilar region, 3 days after drug administration (Figure 6). Activated microglia cells (aMGCs) were classified based on the morphology and size of the cell bodies (Davis et al., 2017). Although a qualitative increase of aMGCs could be observed in the CA1/CA3 regions of both hippocampi of KA- and PL-injected animals (data not quantified), the same phenomenon was observed exclusively in the DG of KA-injected animals. Quantification of aMGCs in the DG revealed a significant increase of aMGCs both in the ipsilateral (86% aMGCs) and contralateral DG (61% aMGCs) of KA-treated animals compared to control and PL-animals (Figures 6L,M; *n* = 3 animals per group; total number IBA1+ cells in ipsi and contralateral sides, respectively: Vehicle – 367 and 336; KA – 241 and 186; PL – 103 and 77). In contrast, only a small, statistically non-significant fraction of aMGCs was observed bilaterally after PL injection (20 and 11% in the ipsi and contralateral DG, respectively), which could indicate the presence of a subtler inflammatory response compared to KA-treated animals. These observations indicate a coincidence between increased proportions of aMGCs and boosted astrogliogenesis or neurogenesis in the ipsi or contralateral sides of KA-injected animals, respectively. It also indicates that increased neurogenesis (observed in the contralateral side of KA animals and both sides of PL animals) can coexist with different degrees of microglial activation in the DG.

## Discussion

**FIGURE 7:**
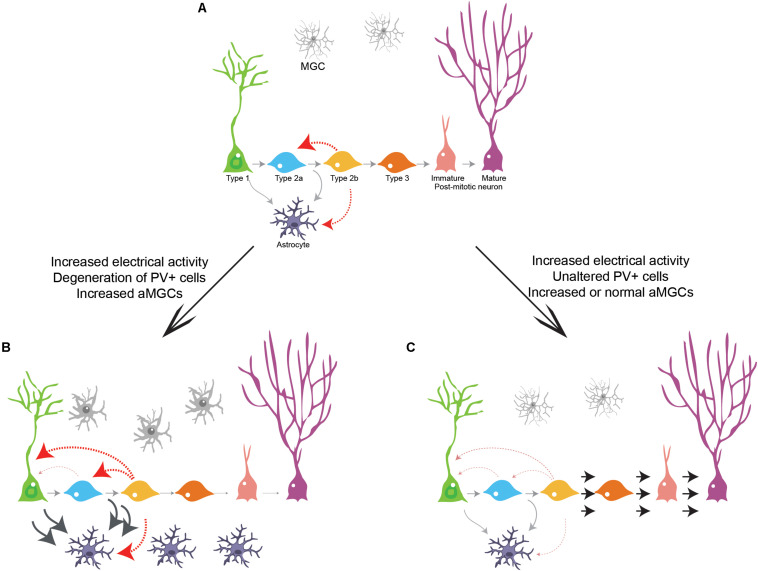
Cell lineage progression model in the adult hippocampus. **(A)** Gray arrows indicate previously described transitions during cell lineage progression from RGC to neurons and from RGC to astrocytes in the adult hippocampus. Red arrows indicate the novel transitions described in our work. **(B)** Enhanced electrical activity accompanied by loss of GABAergic interneurons inhibits the progression of intermediate progenitors toward neurogenesis and stimulate proliferation, generation of RGCs and astrogliogenesis in an environment surrounded by aMGCs. **(C)** Enhanced electrical activity and sustained GABAergic activity stimulate proliferation of intermediate progenitors and neuronal differentiation/survival both in the presence (KA contralateral DG) or absence (PL ipsi and contralateral DG) of aMGCs.

Comprehensive knowledge about the intermediate steps enacted in the transition from an adult neural stem cell to a mature neuronal state is critical to understand how neurons can be generated in the adult central nervous system. Here, we provide evidence that neurogenic cell lineage progression in the adult hippocampus is not a one-way process toward granule cell differentiation. Rather, intermediate progenitors retain the potential to respond to electrical and chemical stimuli, regress to more primitive stages in the cell lineage and regain astrogliogenic potential. Interestingly, increased electrical activity induced by KA injection affects Dcx-cell lineage progression in opposite directions coinciding with distinct alterations in the parvalbuminergic plexus. While degeneration of GABAergic PV+ interneurons coincides with an increase in astrogliogenesis in the ipsilateral DG, preserved numbers of hilar GABAergic PV+ interneurons concurs with an increase in neurogenesis in the contralateral DG. A similar correlation is also observed in both the ipsi and contralateral DG of PL-injected animals, further supporting the notion that neuronal network activity influences intermediate progenitors and thus may control adult neurogenesis in the hippocampus (Deisseroth et al., 2004). We propose that DCX+ intermediate progenitors may function as sensors of global network activity and, therefore, serve as checkpoints for population lineage progression in the adult DG (Figure 7).

Lineage progression in the adult hippocampus has been extensively studied in the last two decades using BrdU-chasing paradigms, viral-mediated, and Cre-Lox fate mapping (Aimone et al., 2014, and references therein). These studies have shown that RGCs (type 1 cells) express GFAP, BLBP, NESTIN, SOX2, and MASH1, are slow-dividing cells, and can also be found in a quiescent state (Song et al., 2012). RGCs function as the founders of the neuronal lineage, generating intermediate progenitors (type 2 cells), which in turn generate type 3 cells, constrained to a future neuronal fate and expressing TBR2, DCX, and NEUROD1 (Suh et al., 2007; Bonaguidi et al., 2011). Some authors also divide type 2 cells into two subtypes - 2a and 2b, where type 2a cells still express several markers of RGCs (BLBP, SOX2, and NESTIN) but do not show radial morphologies (Steiner et al., 2006). In contrast, type 2b express a similar set of proteins to type 3 cells and are considered neuronal-committed progenitors. According to this gene expression pattern, we show that more than 94% of GFP+ cells in the Dcx-lineage are post-mitotic DCX+ immature neurons, and 6% are proliferating DCX+ cells 3 days after tamoxifen administration in the cDcx/GFP mice. These observations are in accordance with previous data in the literature using cDcx mice (Zhang et al., 2010) or immunocytochemistry for DCX (Brown et al., 2003; Jagasia et al., 2009; Liu et al., 2015), and indicate that recombination is mostly restricted to type 2b/3 cells and immature neurons.

Only one single GFP+ cell was observed to express the astrocyte protein GFAP 3 days after recombination. Together with our observations in the SVZ-RMS-OB system, this observation suggests that the ectopic recombination of GFAP-expressing cells is unlikely, since we failed to detect any proliferating GFP+/GFAP+ in control mice 3 days after recombination. Thus, it is reasonable to speculate that at least some of the GFAP+ cells observed in the Dcx-lineage 7 and 30 days after recombination could be generated directly or indirectly from Dcx-progenitors. These evidences may indicate that type 2a/b progenitors are either bipotent or could regress to a more primitive stage in the lineage (type 1/2a), when the astrogliogenic potential is recognized (Bonaguidi et al., 2011; Encinas et al., 2011). According to this latter possibility, we show that a few GFP+ cells in the SGZ maintain morphologies similar to RGL and express GFAP 30 days after recombination. The frequency of these GFP+ RGL cells is significantly increased in the DG of mice receiving KA, where we also observe an increase in astrogliogenesis. Thus, our fate-mapping experiments indicate that at least some type 2b/3 cells do not follow a neurogenic fate, but rather return to stages in the lineage retaining astrogliogenic potential.

Neuronal activity in the hippocampus modulates progenitor cell proliferation, neuronal differentiation, and survival of newly generated granule cells [reviewed by Aimone et al. (2014)]. Drug-induced SE is a well-validated model of human MTLE in which sustained and long-lasting epileptiform activity is used to trigger abnormal network reorganization (Löscher, 2002). While KA binds to kainate receptors leading to membrane depolarization and increased firing activity of principal cells and interneurons (Ben-Ari and Cossart, 2000), PL activates M1 muscarinic receptors resulting in an imbalance between excitation and inhibition (Smolders et al., 1997). We took advantage of these two different mechanisms of action to study how enhanced network activity could affect the cell lineage progression of DCX-expressing cells. Although unilateral intrahippocampal administration of KA or PL acutely elicit SE (Sperk et al., 1983; Turski et al., 1983; Kralic et al., 2005; Heinrich et al., 2006; Ledergerber et al., 2006; Nitta et al., 2008; Häussler et al., 2012; de Lima et al., 2016; Moura et al., 2019) these two neurotoxins have very different effects on Dcx-lineage progression. While PL injection boosted neurogenesis in both ipsi and contralateral sides, KA injection provoked a dramatic shift in the lineage of DCX-cells toward the generation of astrocytes, especially in the ipsilateral side (Moura et al., 2019). Interestingly, despite the fact that neuronal activity is abnormally increased in the contralateral side of KA injection, DCX-cells not only normally progressed to a neuronal fate in this side but also generate a larger number of neurons. These observations are in agreement with previous findings in the literature using BrdU and intrahippocampal KA injection (Kralic et al., 2005; Heinrich et al., 2006; Ledergerber et al., 2006; Nitta et al., 2008; Häussler et al., 2012) or intraperitoneal PL injection (Parent et al., 1997; Scharfman et al., 2000).

Collectively, these data suggest that increased network activity can modulate Dcx-lineage in opposite directions – neurogenesis and astrogliogenesis, depending on the presence of other concomitant signals (see below). Although the effect on neurogenesis can be largely explained by a survival effect on post mitotic neurons (the majority of recombined cells in our experiments), the same is not true for the increased astrogliogenesis observed in the ipsilateral DG of KA animals. The most parsimonious explanation for these findings is that local KA induces a fate switch in the Dcx-lineage. According to this possibility, it has been shown that intrahippocampal KA injection shifts the lineage of NESTIN-expressing cells toward an astroglial fate (Sierra et al., 2015). Although these authors have not documented the indirect effects of KA in the contralateral DG, they have shown that a lower concentration of KA is not sufficient to elicit astrogliogenesis (Sierra et al., 2015). In our study, we used the same KA concentration capable of driving the Nestin-cell lineage toward astrogliogenesis, but we fate mapped progenitor cells at later stages of the lineage progression, previously considered committed to a neuronal fate. We propose that at least some of the Dcx-expressing type 2b/3 progenitors fate mapped in our study could be reversing to a NESTIN+ type 2a and even to a type 1/RGC state in KA-injected animals, thus becoming endowed with an astrogliogenic potential (Figure 7). According to this interpretation, we observe that some cells in the Dcx-lineage show RGC-like morphologies in KA treated animals 3 days after injection. Alternatively, type 2b/3 progenitors could become bipotent and give rise to neurons or astrocytes directly after KA injection (Figure 7).

One important question arising from our data is what could explain the contradictory effects of KA and PL on the DG neurogenesis. The correlation between numbers of GABAergic PV+ cells in the DG and Dcx-cell lineage progression in the ipsi and contralateral hippocampi of KA-injected animals could offer a first cue for a mechanistic explanation to be evaluated in the future. Indeed, while the GABAergic PV+ plexus is mostly preserved in the contralateral side where neurogenesis predominates, the vast majority of GABAergic PV+ neurons acutely degenerate in the ipsilateral side, as previously described (Bouilleret et al., 2000). This loss of PV-expressing neurons in the ipsilateral KA-animals could lead to reduced GABA activity favoring progenitor self-renewal and astrogliogenesis, as shown by previous studies (Song et al., 2012; Dumitru et al., 2017). By contrast, increased network activity in the contralateral KA-injected side and both hippocampi of PL-injected animals could be coupled to an enhanced GABA activity, favoring neuronal differentiation and survival (Jagasia et al., 2009; Bao et al., 2017). In fact, GABA signaling is involved in granule cell survival and differentiation of newly generated DG neurons (Jagasia et al., 2009).

Yet, we cannot rule out the possibility that PL stimulates neurogenesis, at least partially, through the direct activation of muscarinic receptors (Freitas et al., 2006). Depletion of acetylcholine leads to a reduction in neurogenesis (Cooper-Kuhn et al., 2004; Kaneko et al., 2006), whereas increased cholinergic neurotransmission induced by galantamine, an acetylcholinesterase inhibitor, promotes increased neurogenesis (Kita et al., 2014). The latter effect can be partially blocked by scopolamine and the preferential M1 muscarinic receptor antagonist telenzepine (Kita et al., 2014), suggesting that progenitor cells in the hippocampus express muscarinic receptors and are regulated by cholinergic activity.

Another important difference observed in the different SE models used in this study is the pattern of microglial activation/inflammation. Although increased numbers of activated microglia are present in the CA1/CA3 regions of both hippocampi of KA- and PL-injected animals, the same phenomenon was mainly observed in the DG of KA-injected animals. Interestingly, the increased proportion of aMGCs in the ipsi and contralateral DG of KA-animals coincides with opposing effects on the DCX-lineage progression, namely increased astrogliogenesis and neurogenesis, respectively. This coincidence may suggest that inflammation *per se* is not sufficient to change progenitor fate (Bonde et al., 2006; Sierra et al., 2015). Accordingly, controversial reports on the role of inflammation on adult neurogenesis have previously been published: whereas some groups report impaired neurogenesis in the inflamed hippocampus (Ekdahl et al., 2003; Monje et al., 2003), other studies support that inflammation can contribute to the maintenance of neurogenesis (Butovsky et al., 2006). In the future, it would be interesting to modulate inflammation after KA injection to assess possible causal relations between this process and cell lineage progression.

Our results collectively convey a new view on the lineage progression of adult neural stem cells, indicating that intermediate progenitors can regress to more primitive states and switch the potential to generate neurons or glial cells. These alterations in cell-lineage progression concur with microglial activation and enhanced neuronal activity, with GABA potentially playing a key role in the bifurcation between neurogenesis and astrogliogenesis. Last but not least, our conflicting observations for cell lineage progression in two different models of MTLE stresses the need of more studies disentangling the precise mechanisms leading to cellular alterations in the hippocampus of epilepsy patients.

## Data Availability Statement

The raw data supporting the conclusions of this article will be made available by the authors, without undue reservation.

## Ethics Statement

The animal study was reviewed and approved by Comissão de Ética no Uso de Animais – CEUA – UFRN.

## Author Contributions

MC and CQ designed the study. DM and JB performed all experiments using transgenic mice. CL performed the retroviral tracing experiments. MC, CQ, and CH analyzed the data. MC wrote the manuscript with input from all authors.

## Conflict of Interest

The authors declare that the research was conducted in the absence of any commercial or financial relationships that could be construed as a potential conflict of interest.

## Supplementary Material

FIGURE S1 Experimental model for lineage tracing following intrahippocampal injection. **(A)** Mosaic reconstruction of the DG of cDCX/GFP animal 30 d.a.r. showing GFP expression in granule neurons. **(B)** Schematic representation of the intrahippocampal administration of epileptogenic drugs in the right hippocampus. **(C)** Electrophysiological recording during the surgery shows self-sustained Status Epilepticus (SE) in both ipsi and contralateral hippocampi after KA injection. **(D)** Low power image of a coronal section of the mouse brain 30 days after KA injection. Observe the severe granule cell dispersion and CA1 degeneration in the ipsilateral side, whereas the contralateral side histology is mostly preserved. GCL, granular cell layer; CA, *Cornus Ammonis*.

FIGURE S2 Neuronal fate of GFP+ recombined cells 35 days after vehicle or KA injections. Venn diagrams showing the number of GFP+ (green), BrdU+ (red), and CTIP2+ (blue) cells in the ipsi or contralateral hippocampi of 3 controls and 3 KA animals. Observe the small intercept between GFP and BrdU cells in all conditions. Note also the large overlap between GFP and CTIP2 in controls and in the KA contralateral side. Animals received i.p. injections of BrdU 4 times per day (50 mg/kg) for three consecutive days after intrahippocampal injection of KA or vehicle. TAM was also administered i.p. on days 7 and 8. Perfusion and analyses were performed 28 days after TAM (35 days after KA or vehicle injection).

FIGURE S3 Lack of GFP expression in striatal astrocytes following KA injection. **(A)** Timeline representing experimental protocol. **(B)** Schematic view of the KA injection site in the striatum (red dot). **(C–F)** Representative slice showing the absence of GFP+ astrocytes in the striatum 35 days after tamoxifen. **(C)** Confirmation of injection coordinates with DAPI staining. **(E,F)** Enlarged view of merged image. **(E)** Random GFP+ cells can be observed in the striatum confirming the immunohistochemistry. **(F)** GFAP labeling showing reactive astrocytes close to the injection site. **(G–I)** Coronal images of the olfactory bulb showing GFP+ interneurons in the granular and glomerular layers. No astrocytes were observed.

FIGURE S4 DCX+ type 2b cells regress to earlier stages in the progenitor cell lineage. **(A)** Timeline representing experimental protocol in which RGL were found. **(B–D)** Example of RGL-like cells in the DG of cDCX/EGFP that received tamoxifen 35 days before KA injection. Note the typical radial morphology and expression of GFAP in single plane *z*-projection confocal images **(C,D)**, hallmarks of type 1 progenitors. Scale bar: 20 μm.

FIGURE S5 Retroviral-mediated tracing of intermediate progenitors confirm their gliogenic potential. **(A)** Timeline presenting experimental protocol. Adult (P60–90) animals received unilateral ihpc injection of a retrovirus encoding for DsRed fluorescent reporter (pCAG-IRES-DsRed) and 4 weeks later a second ihpc injection of KA or saline (controls). Animals were perfused 4 weeks after the second injection (8 weeks after retrovirus injection). **(B,C)** Coronal sections of the injected DG showing DsRed+ granule neurons in saline-injected controls **(B)** and DsRed+ glial cells in KA-treated animals **(C)**. The white dashed line outline the granule cell layer. **(D)** Quantification of the proportions of DsRed+ granule neurons and DsRed+ glial cells in saline-injected controls versus KA-treated animals. Scale bar: 50 μm.

FIGURE S6 Total number of parvalbuminergic cells in whole hippocampus analysis. Quantification of the number of PV-expressing cells per mm^2^ of the whole dorsal hippocampus including DG and CA regions. [*n* control = 4; *n* KA = 4; *n* PL = 4; Statistics: ANOVA *F* interaction_(2,18)_ = 3.179; *p* = 0.0657; *F* side_(1,18)_ = 14.53; *p* = 0.0013; *F* groups_(2,18)_ = 1.469; *p* = 0.2565; Tukey’s multiple comparisons test DF = 18; Adjusted *p*-value (Ipsilateral control vs. KA) = 0.0418].
