# Supplementary figures and images for: Evidence of Progenitor Cell Lineage Rerouting in the Adult Mouse Hippocampus After Status Epilepticus
Source: Front Neurosci. 2020 Sep 18;14:571315. doi: 10.3389/fnins.2020.571315 (PMC7530340; doi:10.3389/fnins.2020.571315)

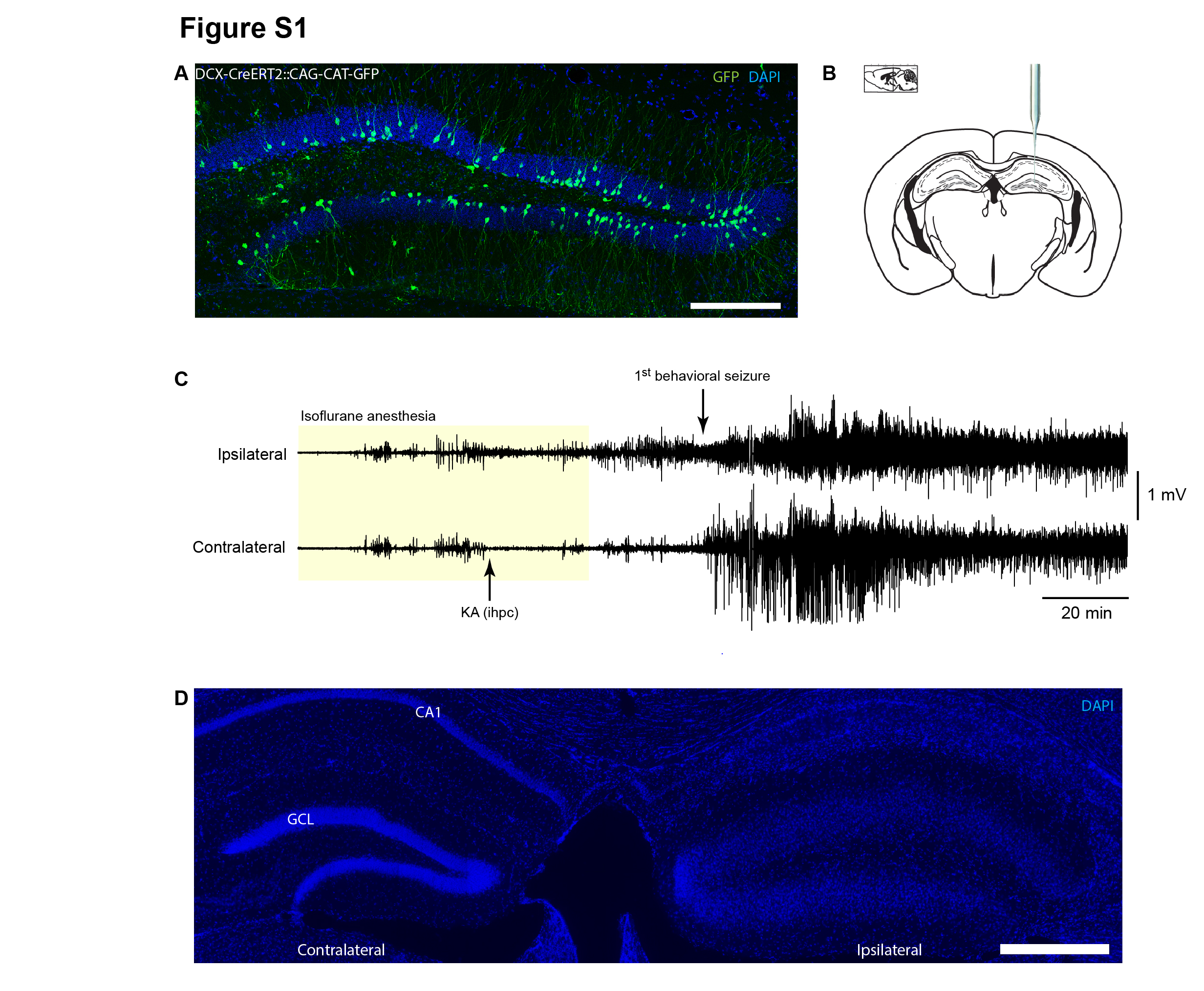

Supplement: FIGURE S1 — Experimental model for lineage tracing following intrahippocampal injection. (A) Mosaic reconstruction of the DG of cDCX/GFP animal 30 d.a.r. showing GFP expression in granule neurons. (B) Schematic representation of the intrahippocampal administration of epileptogenic drugs in the right hippocampus. (C) Electrophysiological recording during the surgery shows self-sustained Status Epilepticus (SE) in both ipsi and contralateral hippocampi after KA injection. (D) Low power image of a coronal section of the mouse brain 30 days after KA injection. Observe the severe granule cell dispersion and CA1 degeneration in the ipsilateral side, whereas the contralateral side histology is mostly preserved. GCL, granular cell layer; CA, Cornus Ammonis. [file Image_1.jpg]

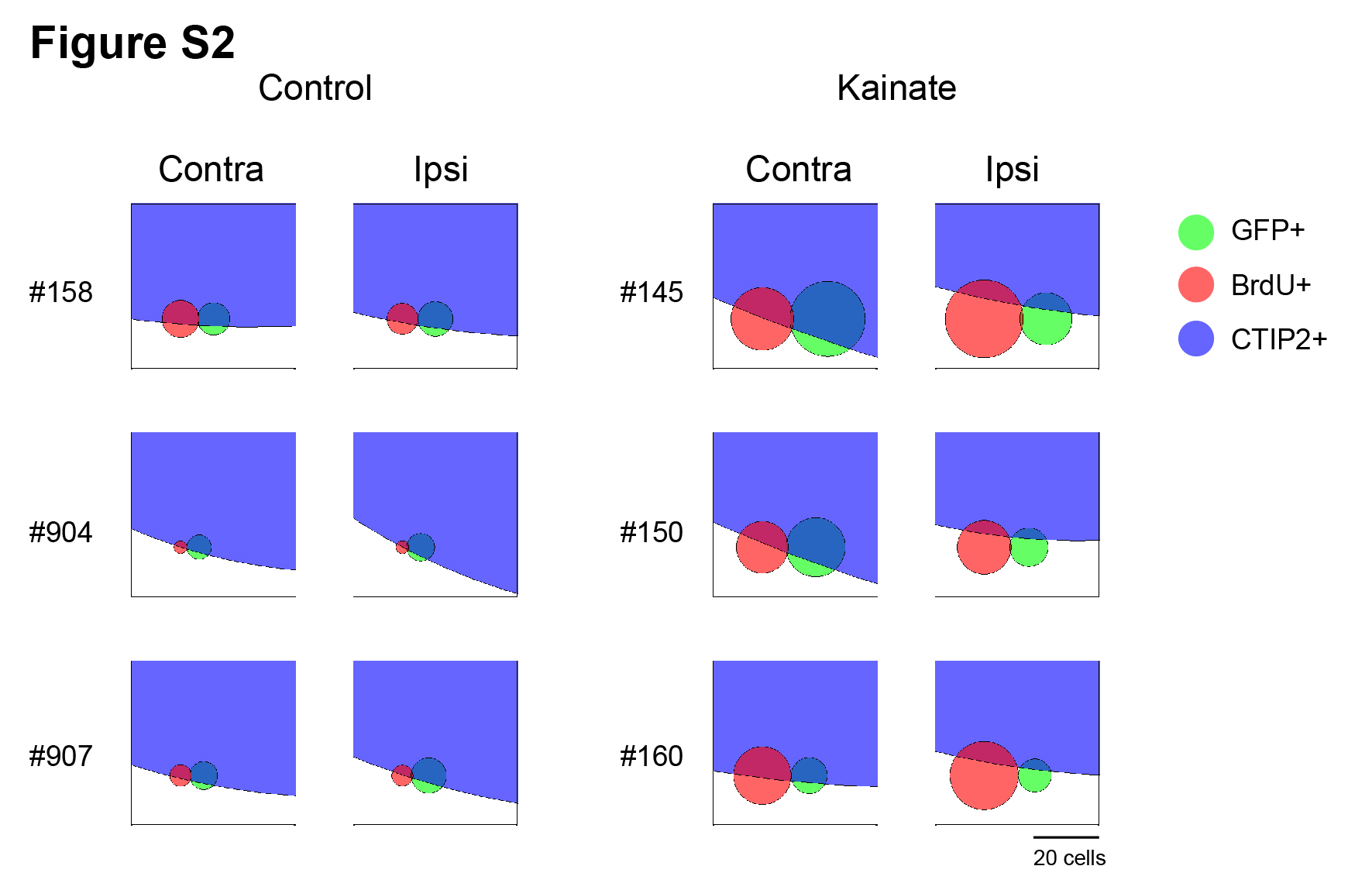

Supplement: FIGURE S2 — Neuronal fate of GFP+ recombined cells 35 days after vehicle or KA injections. Venn diagrams showing the number of GFP+ (green), BrdU+ (red), and CTIP2+ (blue) cells in the ipsi or contralateral hippocampi of 3 controls and 3 KA animals. Observe the small intercept between GFP and BrdU cells in all conditions. Note also the large overlap between GFP and CTIP2 in controls and in the KA contralateral side. Animals received i.p. injections of BrdU 4 times per day (50 mg/kg) for three consecutive days after intrahippocampal injection of KA or vehicle. TAM was also administered i.p. on days 7 and 8. Perfusion and analyses were performed 28 days after TAM (35 days after KA or vehicle injection). [file Image_2.jpg]

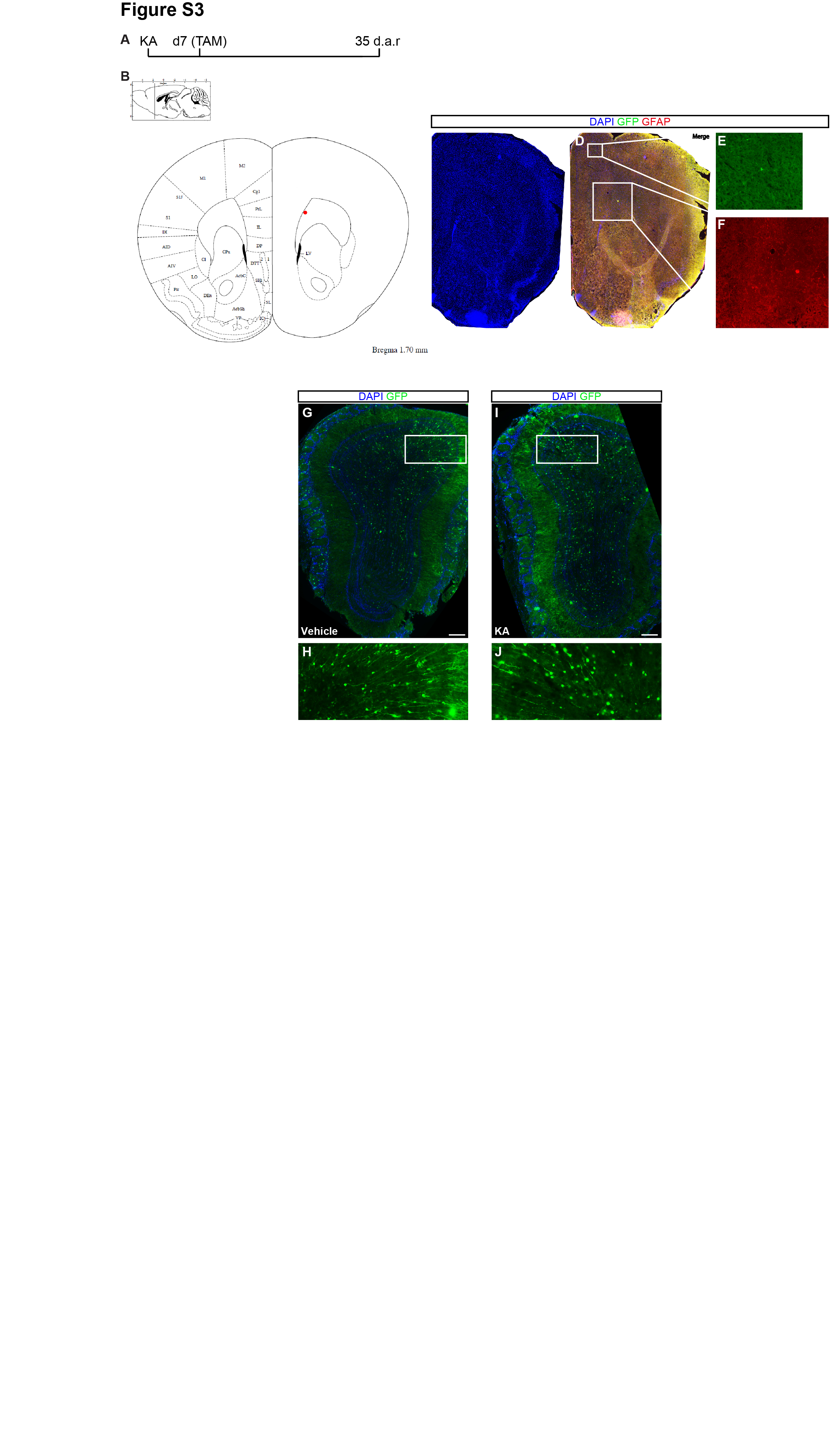

Supplement: FIGURE S3 — Lack of GFP expression in striatal astrocytes following KA injection. (A) Timeline representing experimental protocol. (B) Schematic view of the KA injection site in the striatum (red dot). (C–F) Representative slice showing the absence of GFP+ astrocytes in the striatum 35 days after tamoxifen. (C) Confirmation of injection coordinates with DAPI staining. (E,F) Enlarged view of merged image. (E) Random GFP+ cells can be observed in the striatum confirming the immunohistochemistry. (F) GFAP labeling showing reactive astrocytes close to the injection site. (G–I) Coronal images of the olfactory bulb showing GFP+ interneurons in the granular and glomerular layers. No astrocytes were observed. [file Image_3.jpg]

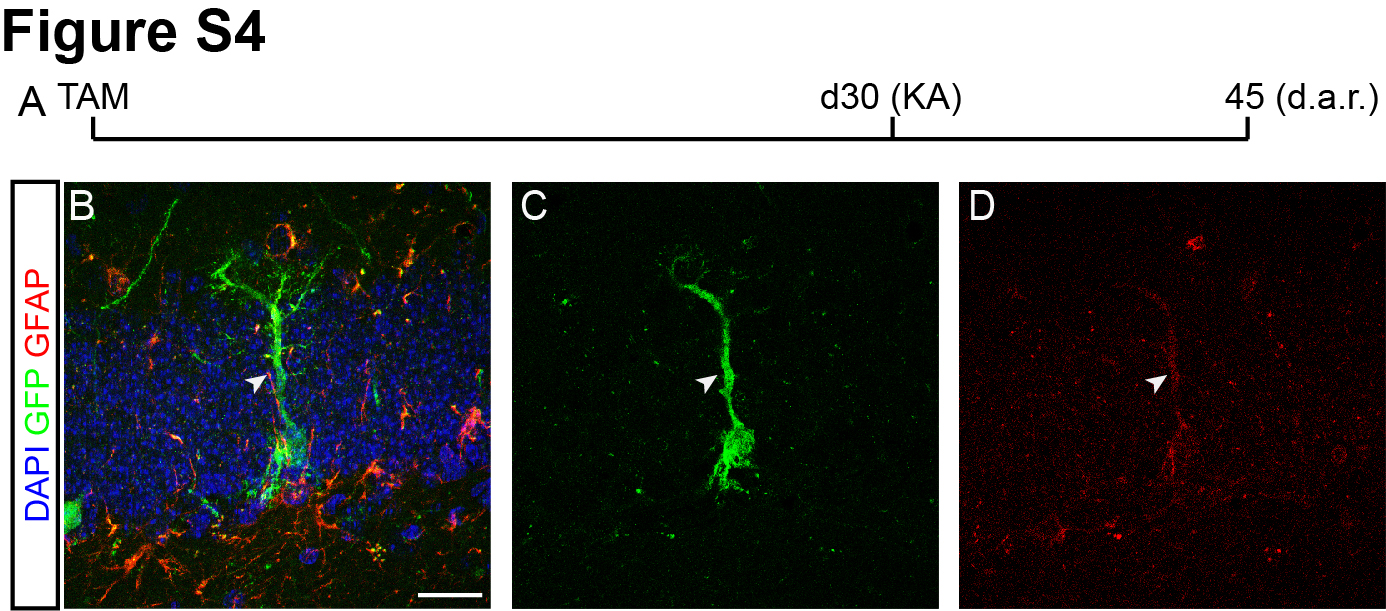

Supplement: FIGURE S4 — DCX+ type 2b cells regress to earlier stages in the progenitor cell lineage. (A) Timeline representing experimental protocol in which RGL were found. (B–D) Example of RGL-like cells in the DG of cDCX/EGFP that received tamoxifen 35 days before KA injection. Note the typical radial morphology and expression of GFAP in single plane z-projection confocal images (C,D), hallmarks of type 1 progenitors. Scale bar: 20 μm. [file Image_4.jpg]

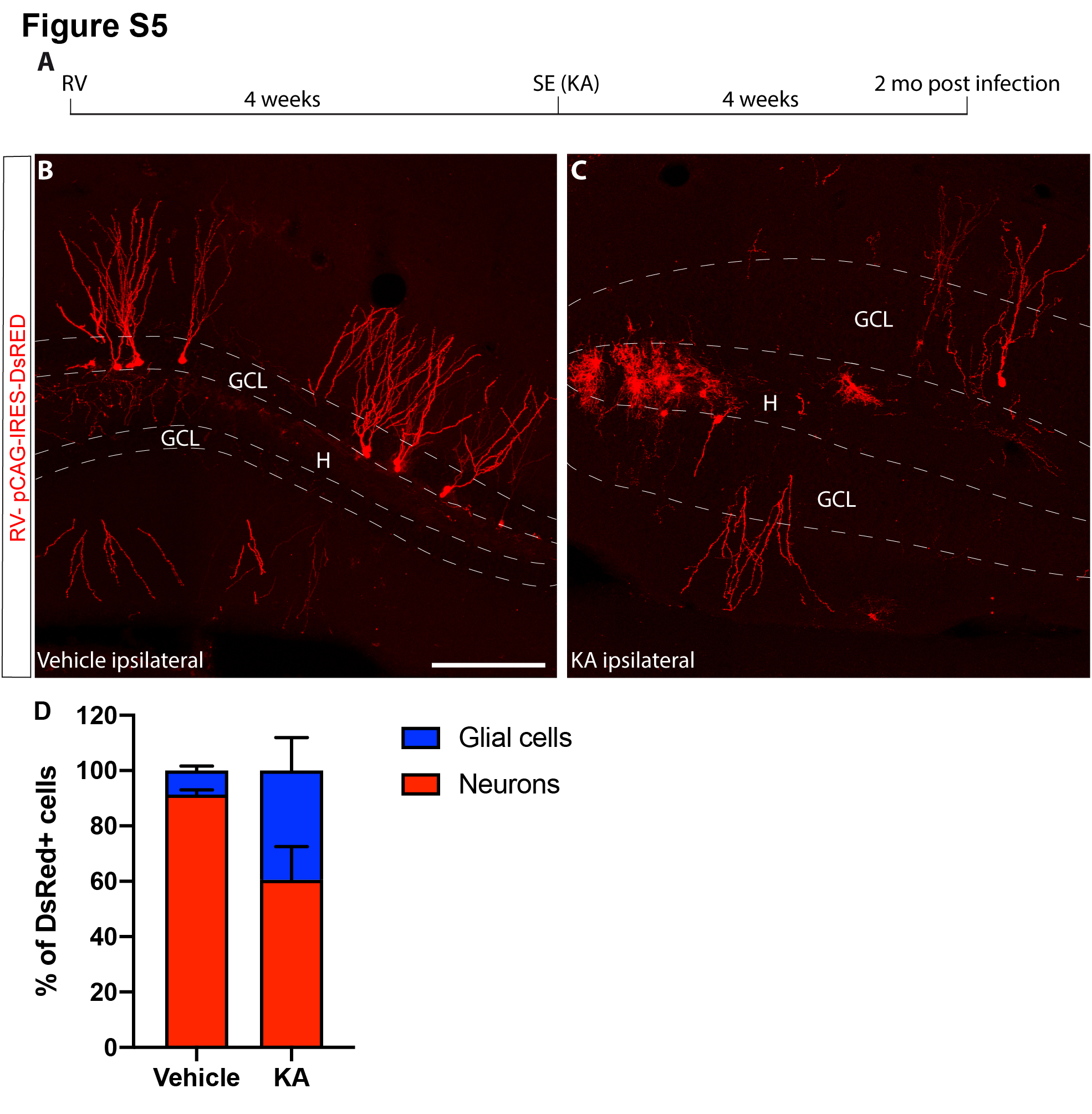

Supplement: FIGURE S5 — Retroviral-mediated tracing of intermediate progenitors confirm their gliogenic potential. (A) Timeline presenting experimental protocol. Adult (P60–90) animals received unilateral ihpc injection of a retrovirus encoding for DsRed fluorescent reporter (pCAG-IRES-DsRed) and 4 weeks later a second ihpc injection of KA or saline (controls). Animals were perfused 4 weeks after the second injection (8 weeks after retrovirus injection). (B,C) Coronal sections of the injected DG showing DsRed+ granule neurons in saline-injected controls (B) and DsRed+ glial cells in KA-treated animals (C). The white dashed line outline the granule cell layer. (D) Quantification of the proportions of DsRed+ granule neurons and DsRed+ glial cells in saline-injected controls versus KA-treated animals. Scale bar: 50 μm. [file Image_5.jpg]

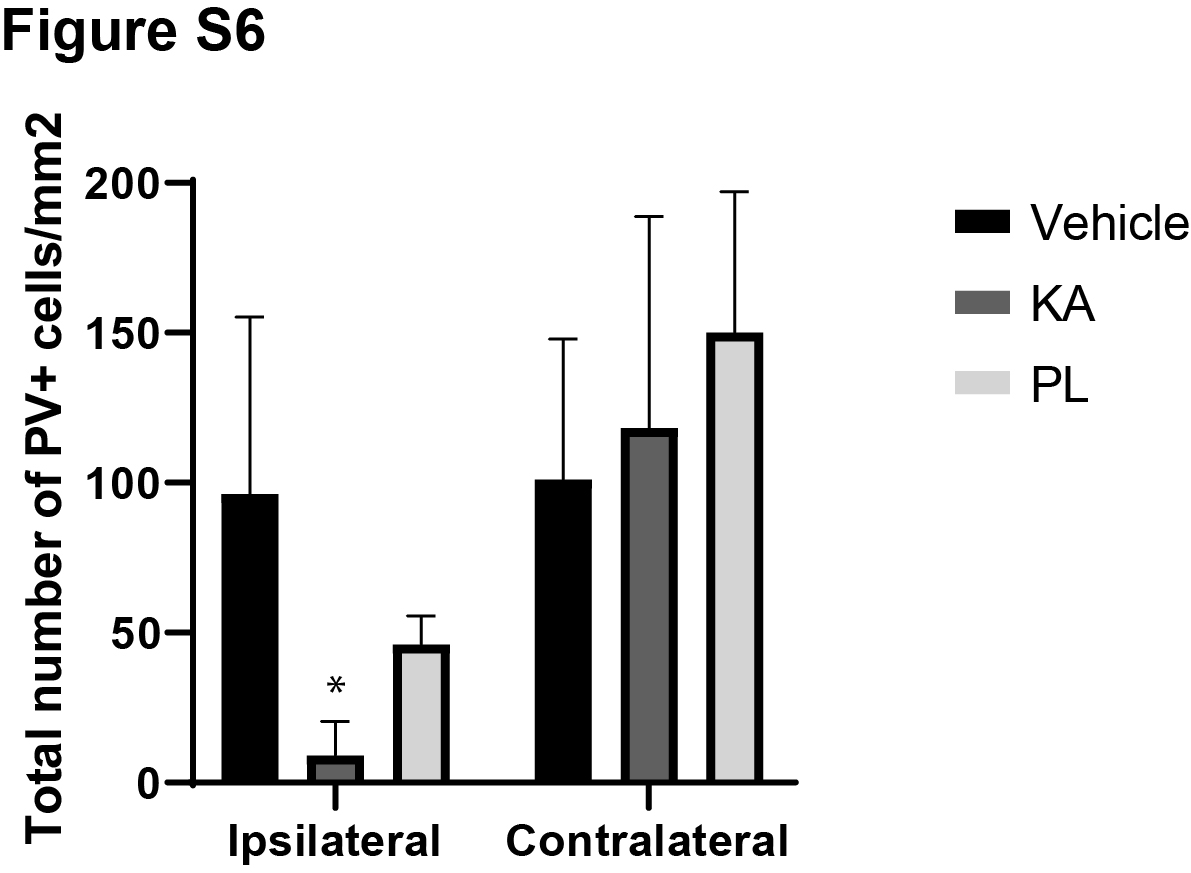

Supplement: FIGURE S6 — Total number of parvalbuminergic cells in whole hippocampus analysis. Quantification of the number of PV-expressing cells per mm2 of the whole dorsal hippocampus including DG and CA regions. [n control = 4; n KA = 4; n PL = 4; Statistics: ANOVA F interaction(2,18) = 3.179; p = 0.0657; F side(1,18) = 14.53; p = 0.0013; F groups(2,18) = 1.469; p = 0.2565; Tukey’s multiple comparisons test DF = 18; Adjusted p-value (Ipsilateral control vs. KA) = 0.0418]. [file Image_6.jpg]
